# Assessment of the Burden of SARS-CoV-2 Variants of Concern Among Essential Workers in the Greater Toronto Area, Canada

**DOI:** 10.1001/jamanetworkopen.2021.30284

**Published:** 2021-10-19

**Authors:** Zain Chagla, Huiting Ma, Beate Sander, Stefan D. Baral, Gary Moloney, Sharmistha Mishra

**Affiliations:** 1 MAP Centre for Urban Health Solutions, St Michael’s Hospital, University of Toronto, Toronto, Ontario, Canada; 2 Institute of Health Policy, Management and Evaluation, University of Toronto, Toronto, Ontario, Canada; 3 Division of Infectious Diseases, Department of Medicine, University of Toronto, Toronto, Ontario, Canada; 4 University Health Network, University of Toronto, Toronto, Ontario, Canada; 5 Department of Epidemiology, Johns Hopkins School of Public Health, Baltimore, Maryland; 6 Department of Medicine, McMaster University, Hamilton, Ontario, Canada

## Abstract

This cohort study examines the burden of SARS-CoV-2 variants of concern among frontline essential workers and by income in the City of Toronto and Region of Peel, Canada.

## Introduction

Variants of concern (VOC) of SARS-CoV-2 emerged toward the end of 2020. These resulted in documented replacement of wildtype SARS-CoV-2, with concerns of transmissibility, virulence, and immune escape, and were subsequently classified by an alphabetical system by the World Health Organization.^1^ Prior to the emergence of VOC, across countries, SARS-CoV-2 transmission and COVID-19 were disproportionately concentrated in neighborhoods of low socioeconomic status, which are characterized by a larger proportion of frontline essential workers, and higher density contact networks.^2,3^ We created a retrospective cohort of neighborhoods in the City of Toronto and Region of Peel, two of the most populous and most affected per capita regions of Ontario,^4^ stratified by income and essential work status, and noted the emergence of VOC through this population.

## Methods

This cohort study was approved by the University of Toronto Health Sciences Research Ethics Board and participant consent was waived because this was a secondary analysis of preexisting health administrative data. This study followed the Strengthening the Reporting of Observational Studies in Epidemiology (STROBE) reporting guideline.

**Figure 1.**
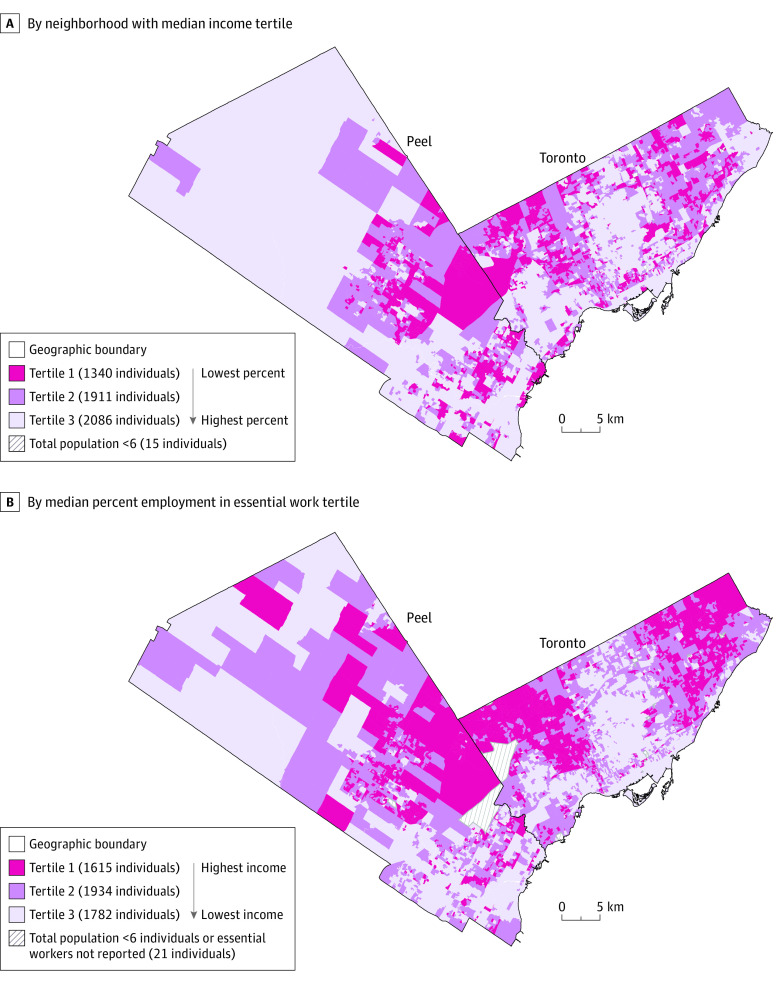
Region of Peel and City of Toronto by Neighborhoods With Median Income Tertiles and Median Percentage Employment in Essential Work Tertiles A, Median income tertiles were tertile 1: $33 819 (IQR, $29 337-37,054); tertile 2: $45 487 (IQR, $41 411-48,079); and tertile 3: $60 652 (IQR, $55 001-69,982). B, Median percentage employment in essential work tertiles were tertile 1: 63.2% (IQR, 59.4%-68.1%); tertile 2: 47.9% (IQR, 42.1%-52.0%); and tertile 3: 30.4% (IQR, 25.0%-35.3%).

We performed a retrospective cohort study on the burden of COVID-19 and VOC characterizing by essential work and income status, using Statistics Canada 2016 Census (self-reported) data for neighborhood-level characteristics, using dissemination areas of approximately 400 to 700 persons. Neighborhoods in the City of Toronto and Region of Peel were stratified into ascending tertiles 1, 2, and 3, which represent 33% of the population ranked by income (where tertile 1 is the lowest income), and essential work status (where tertile 1 is the highest proportion of essential work) represented in Figure 1. Essential work status included the proportion of the working population engaged in essential services (manufacturing, utilities, trades, transport, equipment, agriculture, sales, services, and health). We excluded cases among residents of long-term care homes because they represented a different outbreak setting from community-level outbreaks and transmissions.

We used a public health reportable disease database of person-level data among laboratory-confirmed COVID-19 cases between February 3 and March 10, 2021, and the Statistics Canada 2016 Census data for neighborhood-level characteristics. Polymerase chain reaction (PCR)–positive tests with a cycle threshold less than 35 underwent secondary PCR for the N501Y variation, and genetic sequencing for positive results by centralized laboratories,^5^ representing VOC cases. Of the samples positive for the N501Y mutation, 93% were clade B.1.1.7.^5^ Self-reported race-based data among individuals diagnosed with COVID-19 were collected after June 2021 but were not available for analyses in this study. During the observation period, both regions had closed nonessential businesses, restaurants, fitness centers, and had limits to only interact with one’s household.^6^ Tertiles were compared for case growth during the study period with descriptive analysis conducted in R version 4.0.2 (R Project for Statistical Computing) from February to April 2021.

## Results

**Figure 2.**
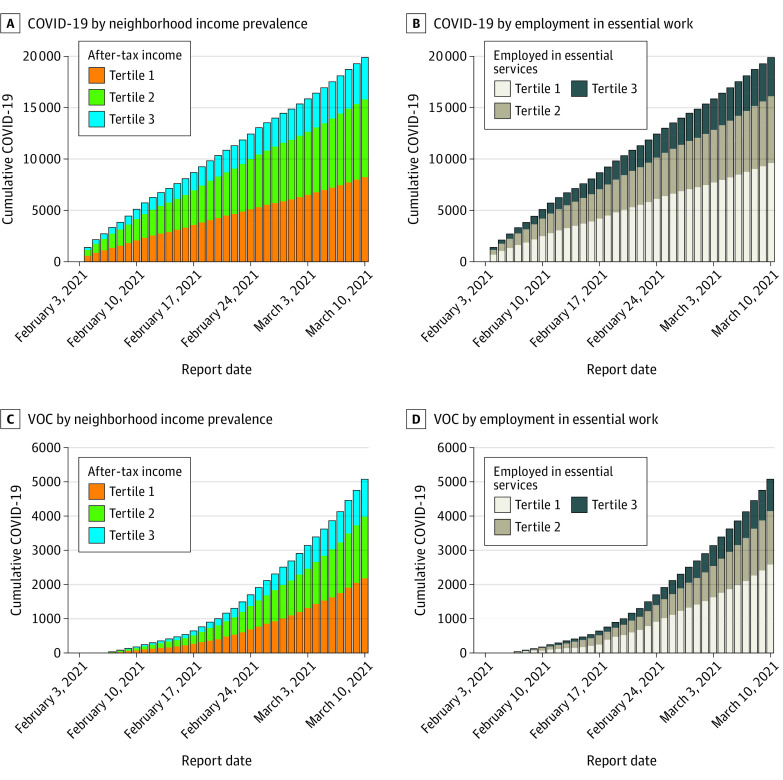
Cumulative COVID-19 Overall and Variants of Concern (VOC) Cases by Neighborhood-Level Prevalence of Income and by Essential Work in Toronto and Peel Regions, Canada

A total of 19 912 COVID-19 cases were observed during the study period, of which 12 860 (64.6%) were screened for a VOC and 5084 (25.5%) screened positive. A similar pattern was observed with income tertiles. Of total cases within the study period, 8723 (43.8%) were in tertile 1, 7085 (35.6%) were in tertile 2, and 4104 (20.6%) were in tertile 3 (Figure 2A); whereas of VOC cases, 2228 (43.8%) were in tertile 1, 1757 (34.6%) were in tertile 2, and 1099 (21.6%) were in tertile 3 (Figure 2C).

When looking at essential work tertiles of total cases, 9597 (48.3%) were in tertile 1, 6504 (32.7%) were in tertile 2, and 3788 (19.0%) were in tertile 3 (Figure 2B). Among VOC cases in essential work tertiles, 2582 (50.8%) were in tertile 1, 1562 (30.8%) were in tertile 2, and 934 (18.4%) were in tertile 3 (Figure 2D).

## Discussion

These findings suggest that VOC of SARS-CoV-2, similar to wildtype SARS-CoV-2, are disproportionately associated with neighborhoods with lower income and a higher proportion of essential workers.^2^ Notably, these analyses have some limitations. Use of ecological study design, rather than individual-level data on income and occupation for this analysis; and the potential for differential SARS-CoV-2 testing across tertiles, particularly less testing in low-income neighborhoods and essential work communities may result in underreporting both wildtype and VOC cases. Finally, not all samples were sent for VOC testing, and although testing was centralized, we cannot accurately ascertain if there were testing differences by region, with potential for a selection bias. Given the rapid mirroring of wildtype epidemics, these results suggest an association between essential work, income, and COVID-19 burden, which may be magnified by more transmissible variants. Reducing SARS-CoV-2 transmission and the associated morbidity and mortality necessitates tailored and equitable intervention strategies including vaccine prioritization and outreach services.

## References

[1] World Health Organization. Tracking SARS-CoV-2 variants. Accessed July 2, 2021. https://www.who.int/en/activities/tracking-SARS-CoV-2-variants/

[2] Sundaram M, Calzavara A, Mishra S, The individual and social determinants of COVID-19 diagnosis in Ontario, Canada: a population-wide study. medRxiv. Preprint posted online March 21, 2021. doi:10.1101/2020.11.09.20223792 PMC8177943 33906966

[3] Rüdiger S, Plietzsch A, Sagués F, Sokolov IM, Kurths J. Epidemics with mutating infectivity on small-world networks. Sci Rep. 2020;10(1):5919. doi:10.1038/s41598-020-62597-5 32246023 PMC7125191

[4] Public Health Ontario. Weekly epidemiologic summary COVID-19 in Ontario: Focus on March 7, 2021 to March 13. Published March 2021. Accessed September 17, 2021. https://files.ontario.ca/moh-covid-19-weekly-epi-report-en-2021-03-13.pdf

[5] Public Health Ontario. SARS-CoV-2 (COVID-19 Virus) variant of concern (VoC) surveillance. Updated March 22, 2021. Accessed September 17, 2021. https://www.publichealthontario.ca/en/laboratory-services/test-information-index/covid-19-voc

[6] Government of Ontario. COVID-19 public health measures and advice. Updated March 19, 2021. Accessed September 17, 2021. https://covid-19.ontario.ca/public-health-measures

